# Therapeutic Notes

**Published:** 1898-10

**Authors:** W. J. Martin

**Affiliations:** Kankakee, Ill.


### THERAPEUTIC NOTES.

UNDER THE DIRECTION OF W. J. MARTIN, M.D.C.,
KANKAKEE, ILL.

FORMALDEHYDE DISINFECTION WITHOUT APPARATUS.—The Chicago Health Department claims to have obtained better results in recent municipal disinfection by the use of this agent, without any apparatus, than heretofore with the various autoclaver generators and other devices. Ordinarily these, suspended in the room, were simply sprayed with the 40 per cent. solution through a common watering-pot rose-head. A sheet of the usual size and quality will carry from 150 to 180 c.c. of the solution without dripping, and this quantity has been found sufficient for the efficient disinfection of a thousand cubic feet of space. Of course, the sheets can be multiplied to any necessary number.

Cultures, both moist and dry, were exposed for five hours in these experiments, some in sealed envelopes and others wrapped in three thicknesses of sheets, or wrapped inside of woollen blankets. Of the former, none showed growth after seventy-two hours incubation, while the growth was but slight in those wrapped in the blankets. Surface disinfection was thorough, while a much greater degree of penetration was shown in these experiments than that secured by any other method.

The evolution of the gas from the sprinkled sheets is exceedingly rapid, so much so that it behooves the operator to vacate the room within a very few seconds, while after starting the ordinary generator he may remain ten minutes or more without serious inconvenience. When the room is opened, after five hours, the density of the gas is still so great as to preclude respiration until after doors and windows have been opened some little time.

On the other hand, the air is respirable within a very few minutes after the sheet has been removed, and there is no lingering smell of formaldehyde for days after, as is the case where the gas is evolved by the action of heat. This is due to the fact that a minimum of the paraform is produced in the evaporation of the solution at the ordinary temperature, and this is retained in the meshes of the fabric instead of being preciptiated on surfaces to be slowly converted into the gaseous forms through several days.

If further experiments which are now being prosecuted by the department shall confirm the results thus far obtained, the problem of practical domestic disinfection by formaldehyde would seem to be in a fair way to be solved.—*Therapeutic Progress.*

ANTIDIPHTHERIA SERUM IN ASTHMA.—Injections hypodermatically of antidiphtheria serum have been successfully employed in relieving and curing asthma in man.

HOOF OINTMENT.—Neatsfoot-oil, 1 quart; mutton-tallow, ½ pound; yellow wax, ½ pound; castor-oil, 1 pint; lampblack, ½ ounce. Mix by aid of a gentle heat. This is an excellent preparation to use in contracted heels, corns, quarter-cracks, etc.; it may be used every other day.

VETERINARY FLY-BLISTER.—Cantharides in powder, ar., ʒijss; oil turpentine, flʒjss; acetic acid, flʒj; wool-fat, ar., ʒv; petrolatum, ar., ʒv. Mix the first three ingredients and allow to stand for twenty-four hours; then add the wool-fat and petrolatum, previously mixed in a water-bath, and mix, stirring until cold.—*Western Druggist.*

ANTIPYRINE IN SUNSTROKE.—Antipyrine has been successfully employed hypodermatically in treating sunstroke in man. This remedy might also be used with great benefit in treating sun- or heatstroke occurring in horses. A solution containing from 40 to 80 grains to 2 drachms of distilled water, ready for instant use, could be easily carried in the medicine-case during hot weather.

---

AN ANCIENT HOSPITAL.—Recently, while archæological excavations were being carried on at Baden, Switzerland, the remains of a building that had been used by the ancient Romans as a hospital was discovered. In the ruins were found many medical instruments and utensils, such as probes, tubes, pincers, cauterizing instruments, safety-pins, medicine spoons of bone, silver measuring-vessels, jars and pots for medicines, some even containing traces of the ointments and medicaments which they once contained. There were fourteen rooms in the building.

